# Optical Properties of Transparent Rare-Earth Doped Sol-Gel Derived Nano-Glass Ceramics

**DOI:** 10.3390/ma14226871

**Published:** 2021-11-14

**Authors:** Mihail Secu, Corina Secu, Cristina Bartha

**Affiliations:** National Institute of Materials Physics, 077125 Magurele, Romania; cesecu@infim.ro (C.S.); cristina.bartha@infim.ro (C.B.)

**Keywords:** sol-gel, glass ceramic, nanocrystals, fluorides, rare-earth, luminescence

## Abstract

Rare-earth doped oxyfluoride glass ceramics represent a new generation of tailorable optical materials with high potential for optical-related applications such as optical amplifiers, optical waveguides, and white LEDs. Their key features are related to the high transparency and remarkable luminescence properties, while keeping the thermal and chemical advantages of oxide glasses. Sol-gel chemistry offers a flexible synthesis approach with several advantages, such as lower processing temperature, the ability to control the purity and homogeneity of the final materials on a molecular level, and the large compositional flexibility. The review will be focused on optical properties of sol-gel derived nano-glass ceramics related to the RE-doped luminescent nanocrystals (fluorides, chlorides, oxychlorides, etc.) such as photoluminescence, up-conversion luminescence, thermoluminescence and how these properties are influenced by their specific processing, mostly focusing on the findings from our group and similar ones in the literature, along with a discussion of perspectives, potential challenges, and future development directions.

## 1. Introduction

Glass ceramics are inorganic, non-metallic materials prepared by controlled crystallization of precursor glasses with at least one type of functional crystalline phase, that may vary from ppm to almost 100% is embedded in residual glass [1]. The basic requirements for optical transparency include: nanocrystals size smaller than the light wavelength, very close refractive index of amorphous and crystalline phases and low birefringency [1]. Transparent oxyfluoride nano-glass ceramics are produced by fluoride nanocrystals precipitation in a silica glass matrix during a controlled thermal processing. They have shown a high potential for applications in various devices (optical amplifiers, optical waveguides, white LEDs, etc.) since they combine the optical phenomena with optical transparency due to lack of scattering.

The conventional way to obtain glass ceramics is by melt-quenching (at around 1400 °C for oxy-fluoride glasses or a lower temperature in a controlled atmosphere for fluoride glasses) followed by crystallization of the precursor glass during a controlled annealing step throughout a heat-treatment at lower temperatures. Nucleation and growth of fluoride nanocrystals occurs in the glass matrix during heat treatment and rare earth ions can preferentially segregate into the fluoride nanocrystals. Hence, the partition of the optically active rare-earth (RE) ions into the precipitated fluoride nanocrystals can be obtained, with low phonon frequencies, but keeping good chemical and mechanical stability of the oxide glass (see the review of de Pablos-Martin et al. [2]). An alternative route is based on sol-gel chemistry, well known as a flexible approach to preparing various materials such as glass, ceramics, and organic-inorganic hybrids by using colloidal solutions (sol) as starting materials [3,4]. The glass ceramics’ synthesis by using the sol-gel method has several advantages such as lower processing temperature, the ability to control the purity and homogeneity of the final materials on a molecular level, and the large compositional flexibility which could be difficult to obtain via the melt-quenching technique. Most of the sol-gel derived oxyfluoride glass ceramic materials studied have a simple composition according to the formula: (100 − x) SiO_2_-xM1F_2_/M2F_3_/M1M2F_4_, where M1 and M2 are alkaline, alkaline-earth metals, or lanthanide elements, respectively [4].

This review is focused on luminescence properties of sol-gel derived nano-glass ceramics related to the RE-doped luminescent nanocrystals (fluorides, chlorides, oxychlorides, etc.) such as photoluminescence, up-conversion luminescence, thermoluminescence and how these properties are influenced by thermal processing, focusing on the findings from our group and similar ones in the literature of the past decade, along with a discussion of the future perspectives, potential challenges, and future development directions.

## 2. Sol-Gel Method for Glass Ceramic Synthesis

The pioneering contribution on sol-gel derived oxyfluoride glass ceramic preparation came from the first works of Fujihara et al. [5,6,7] performed in two steps: (i) preparation of a silica sol by using classic route with metal alkoxides (ii) followed by mixing with a second trifluoroacetates solution formed by trifluoroacetic (TFA) acid reaction with rare earth precursors (acetates). The liquid mixture is dried and aged at room temperature under sealed containers for several days or weeks. The nanocrystalline phase precipitation is obtained during subsequent calcination of dried xerogel matrix above the temperature required for thermal decomposition of corresponding trifluoracetates at around 300 °C:

RE(CF_3_COO)_3_ → REF_3_ + (CF_3_CO)_2_O + CO + CO_2_ (1)

Since then, the number of the publications on that topic has increased steadily and many compositions and crystalline phases have been studied using different Ln^3+^ ions as dopants to obtain enhanced linear and nonlinear optical processes for a wide application range.

### 2.1. Crystallization Mechanism

The first investigations of the crystallization processes of sol-gel derived glasses were done by Fujihara et al., with MgF_2_ thin films synthesis from trifluoracetic acid (TFA) and Mg-ethoxide (Mg(OC_2_H_5_)_2_ as precursors. It was assumed that the Mg^2+^ ions released during the thermal decomposition of the Mg(OC_2_H_5_)_2_ complexes form the xerogel and react with thermally activated fluorine in CF_3_COO^−^ ions to form MgF_2_ crystals [7].

**Figure 1:**
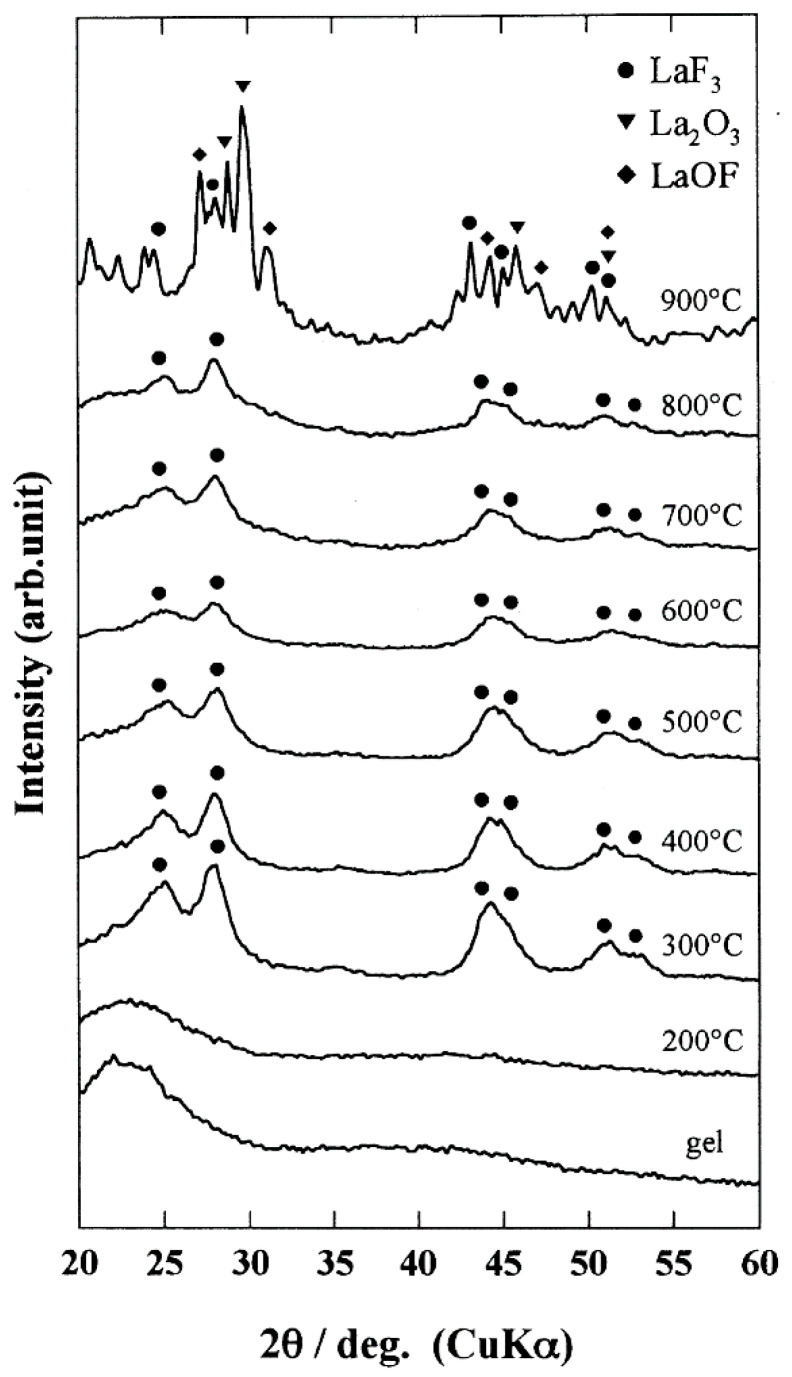
The XRD patterns of SiO_2_–LaF_3_ gel and glass ceramics obtained after calcination at different temperatures. (Reproduced from reference [5].)

The investigations of the SiO_2_–LaF_3_ sol gel system evolution during the heat treatment by using Fourier transform infrared spectroscopy (FTIR) analysis indicated decomposition of trifluoroacetic acid (TFA), used as a fluorine precursor, together with the formation of fluoride lattice bonding [5,6] (Figure 1). The formation of LaF_3_ nanocrystals (of about 10 ± 30 nm in size after calcination above 300 °C of the precursor silica xerogel) was assigned to the screening effect of the CF_3_COO^−^ ions coordinating the La^3+^ ions that prevented the La^3+^ ions incorporation into the SiO_2_ matrix [5]. The smallness of the LaF_3_ nanocrystals (tens of nm size) even for higher temperatures calcination was assigned to the low nanocrystals growth rate because of the uniform distribution of the nanocrystals [5]. For higher annealing temperatures above 800 °C, the formation of LaF_3_, La_2_O_3_, and LaOF nanocrystals was observed [5].

Later on, the studies of the crystallization kinetics of sol gel-derived glasses were extended by using multiple techniques (structural, magnetic, optical), which indicated a decomposition chemical reaction accompanied by the precipitation of nanocrystals, and not a diffusion-controlled nucleation and growing process, as in the melt-quenched glasses [6,7,8,9,10]. For longer treatment times, the loss of crystal structure and a partial/total dissolution of crystals in the glass matrix was observed. Higher annealing temperature does not result in better crystallinity or bigger nanocrystals but for even higher temperatures (1000 °C) the Si–O–Si network is heavily destroyed due to precipitation of the SiO_2_ crystalline phase, so the nano-crystals size grow quickly [11]. It was shown that the nanocrystal growth process in the glass matrix is strongly influenced by the ionic environment and ionic impurities incorporated during the growth process, and energy dispersive X-ray (EDX) analysis indicated the presence of oxygen ions in the nanocrystals [12].

**Figure 2:**
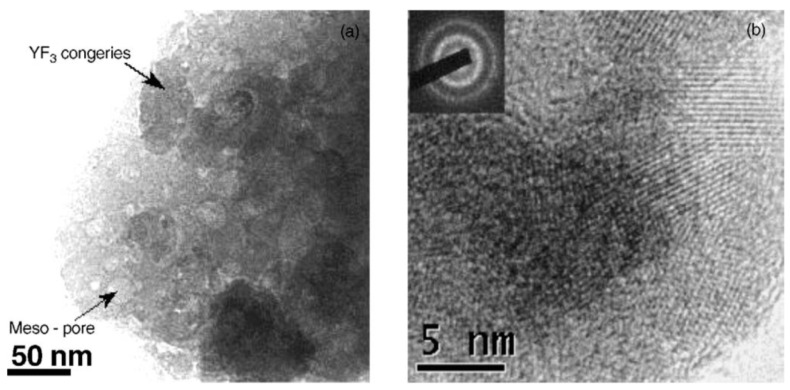
TEM images of the SiO_2_–YF_3_ xerogel sample calcinated at 400 °C (**left**) showing the congeries (**right**) (reproduced from reference [12]).

Structural and morphological studies of the crystallization mechanism [13] of the SiO_2_–YF_3_ glass ceramic showed that the crystallization of YF_3_ nanocrystalline phase is due to decomposition of Y(CF_3_COO)_3_, according to the previous image of the crystallization mechanism. Moreover, transmission electron microscopy (TEM) investigations showed that YF_3_ nanophase crystallization started with formation of big congeries particles in the glassy matrix consisting of many randomly oriented mono-crystallites sized around 5 nm (Figure 2). Higher calcination temperature (up to 600 °C) promotes the separation of the initial nanocrystals by reducing stress and lowering system energy. A similar conclusion was reached by Yunlong Yu et al. (2006) [11], who showed that the formation of SrF_2_ nano-crystals 8–10 nm distributed homogenously among the glassy matrix. The nanocrystals size remains almost unchanged up to 800 °C annealing due to the interfacial interaction of SrF_2_ nano-crystals with the glass matrix, which hinders their further growth.

**Figure 3:**
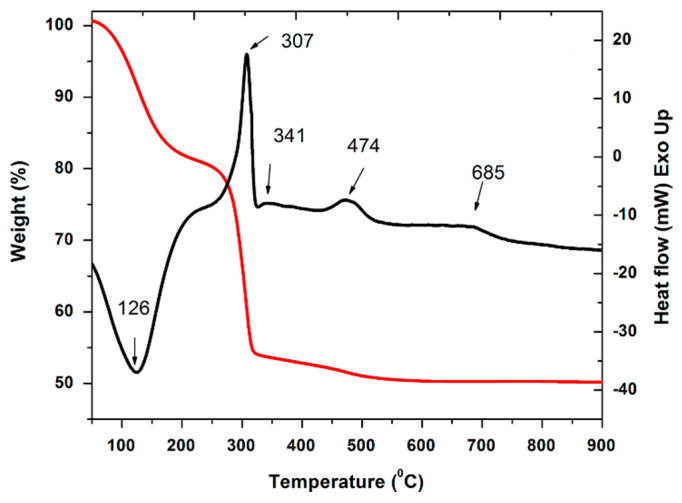
Thermal analysis of Eu-doped SiO_2_–BaF_2_ xerogel showing the TG (red) and DSC (black) curves (reproduced from reference [8]).

Structural and optical investigations of the crystallization process in SiO_2_–BaF_2_ sol-gel indicated a homogenous crystallization mechanism, with BaF_2_ small nanocrystals (a few nm size) acting as nucleation centers that resulted from thermal decomposition of Ba–trifluoroacetate at about 300 °C [8]. The investigations of thermal behavior of the silicate xerogels by using differential scanning calorimetry (DSC) and thermogravimetry (TG) showed a three-stage thermal degradation profile related to the glass ceramization process [8] (Figure 3). The first step corresponding to the temperature range from 120 °C to 200 °C is due to the removal of volatile components. A second step from 280 to 360 °C is closely related to the Ba trifluoroacetate decomposition [14] with the formation of tiny BaF_2_ nanocrystalline seeds (few nm size) and is accompanied by a strong DSC peak at about 307 °C. The next weight loss in the TG curve occurs in the temperature range from 400 to 500 °C and is due to the pyrolysis of organic groups bonded to silicon (residual organics and carbon). The formation of the BaF_2_ nanocrystalline phase in Eu^3+^-doped SiO_2_–BaF_2_ and crystallinity improvement was associated to a broad and weak DSC peak at 685 °C (Figure 3) and shows a slight dependence (within few degrees) on the RE-dopant ions incorporation. A similar peak was reported at 663 °C in 95SiO_2_–5CaF_2_ [15] and at 700 °C in 95SiO_2_–5SrF_2_ [11] xerogel, but not in the undoped one, indicating its strong relation to the nature of the nanocrystalline phase.

Therefore, the general characteristic of the sol-gel glasses crystallization process is the thermal decomposition reaction of the metal trifluoroacetates, revealed as a strong DSC peak between 280 and 350 °C [10,16]. Higher calcination temperature results in a weak increase of the nanocrystals size by promoting the separation and growth of the initial nanocrystals [13]. These processes are revealed by a second and weaker DSC peak, which is strongly dependent of the fluoride nanocrystals nature. However, the mechanism is expected to be more complicated for glass ceramics containing complex compounds such as NaYF_4_, NaLaF_4_, NaGdF_4,_ KLaF_4,_ KYF_4_, LiYF_4_, BaYF_5_, and BaGdF_5_ (refer to the next section).

### 2.2. Optical Properties: Photoluminescence, Up-Conversion Luminescence, and Thermoluminescence

The calcination of dried xerogel affects not only structural and morphological properties (i.e., nanocrystals formation), but also the optical ones. During xerogel calcination, a silica network is formed due to the progressive enhancement of dehydration–condensation reactions [8,15,17], accompanied by the precipitation of the fluoride nanocrystalline phase, where a fraction of the RE^3+^ ions was incorporated. It was shown that in Er^3+^-doped SiO_2_–LaF_3_ sol-gel glass ceramic the effective concentration of rare-earth ions estimated by X-ray absorption spectroscopy was 91% and 9% in a fluoride and glass environment, respectively [18].

**Figure 4:**
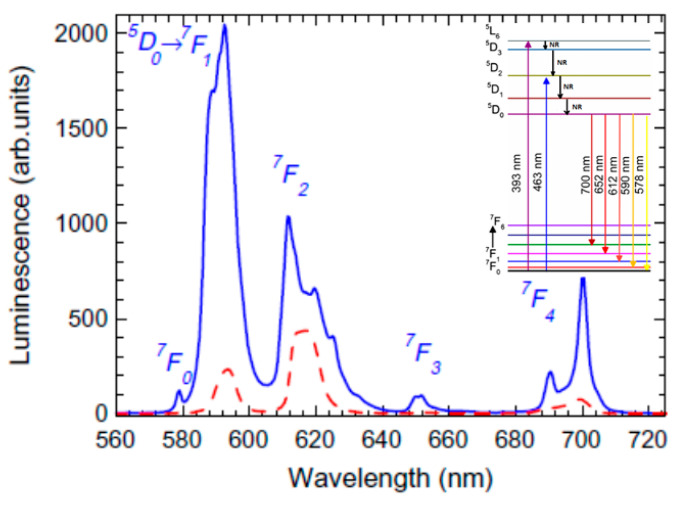
Photoluminescence spectra recorded on Eu^3+^-doped xerogel (dotted curve) and glass ceramic (solid curve) using 394 nm excitation wavelength (modified from reference [19]); the inset shows the Eu^3+^ energy levels diagram.

In the amorphous matrix, the RE^3+^-ions dopants are distributed within the amorphous structure of the glass, characterized by unregular arrangement of the constituent ions. Hence, in the glass matrix, the photoluminescence spectra of the RE^3+^-ions shows broadening due to the random positions and random surroundings of the dopant ions. The new luminescence features observed in the glass ceramic were assigned to the RE^3+^-ions incorporated into the precipitated nanocrystals. The luminescence spectra in the glass ceramic shows Stark splitting due to the degeneracy lifting by the local crystal field where the RE^3+^-ion are incorporated, an effect clearly observed for Eu^3+^ ions [19,20]; Figure 4. Because of the nanocrystals smallness in the glass ceramic, the nanosize-related effects can have a strong influence on the PL spectra (i.e., broadening effects), as was observed in the nanocrystals [21,22]. Moreover, on the nanocrystals surface, we expect a high concentration of surface atoms and defects [21,22] acting as trapping centers and non-radiatively dissipating energy. Therefore, the PL spectra are assigned to a superposition between the RE^3+^ ions emission located in the glass matrix and in the crystalline phase, inside the nanocrystals and on their surface [19]. Photoluminescence decay measurements are also highly sensitive to the RE^3+^-ions environment. Lower phonon energy of fluorides and the dehydration processes reduce the probability of non-radiative de-excitations and as a result a luminescence signal enhancement and longer luminescence lifetimes compared to the xerogels were observed [8,18,23]. Defects with relatively large vibration energies like hydroxyl groups from the water, solvent, and silanol groups are efficient non-radiative relaxation channels for the excited states and therefore multiphonon relaxation rate is rather large, thereby decreasing the luminescence lifetime and efficiency.

**Figure 5:**
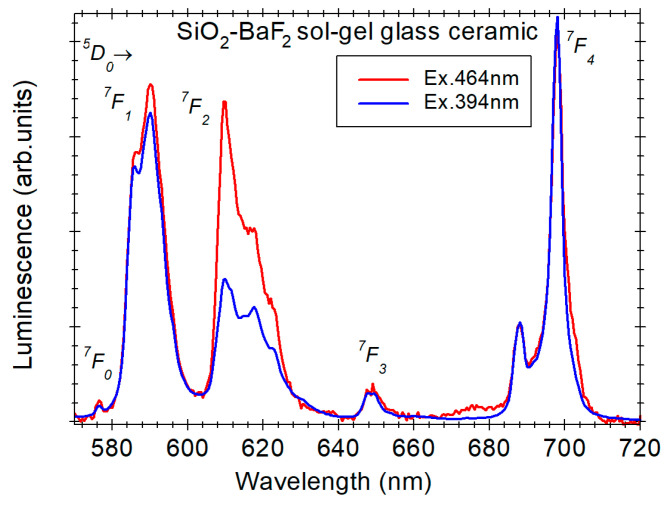
Photoluminescence spectra of Eu (1%)-doped 95SiO_2_–5BaF_2_ nano-glass ceramic (prepared in ref. [8]) recorded under 394 and 464 nm excitation.

Among the RE^3+^ ions, Eu^3+^ ions are widely used as green–red light emitting activators, where the characteristic luminescence properties are to the intra-configurational 4f^6^–4f^6^ transitions (^5^D_0_ → ^7^F_J_, *J* = 0–4); see Figure 4. Moreover, they are widely studied as probe-ions for local site symmetry in various materials [24,25] and have been used to investigate the sol-gel process [8,26,27,28]. The electric-dipole ^7^D_0_ → ^5^F_2_ transition is sensitive to the environment and therefore the intensity ratio between the two visible emissions ^5^D_0_–^7^F_2_/^5^D_0_–^7^F_1_ is highly dependent on the Eu^3+^-ion environment. The lower this ratio, the closer is the local symmetry to the one having an inversion center [29]. In particular, the intensity ratio has been used to monitor and discuss the sol-gel process and xerogel transformation in various Eu^3+^-doped glass ceramics [8,20,23,30]. Moreover, luminescence spectra recorded under proper excitation wavelengths and luminescence decay measurements recorded in SiO_2_–LaF_3_ glass ceramic have allowed discerning between ions residing in precipitated nanocrystals and those remaining in a glassy environment [23]. The 464 nm excitation wavelength is inhibited for Eu^3+^ ions in high symmetry sites, whereas the broad luminescence spectrum and shorter luminescence decay lifetime reflect the glassy environment [23]. By comparison, the luminescence spectra recorded under 394 nm excitation (the ^7^D_0_ → ^5^F_2_ transition) shows Stark splitting and longer decay time characteristic to the crystalline environment [23]. A similar approach was applied to the Eu^3+^-doped SiO_2_–BaF_2_ glass ceramic (prepared in ref. [8]) and the spectra showed structured luminescence spectra for both excitation wavelengths but different red to green visible emissions ratios (Figure 5). The spectra are consistent with dominant incorporation of the Eu^3+^ ions within the BaF_2_ nanocrystals [8], exhibiting two different crystalline sites with higher and lower local symmetry.

**Figure 6:**
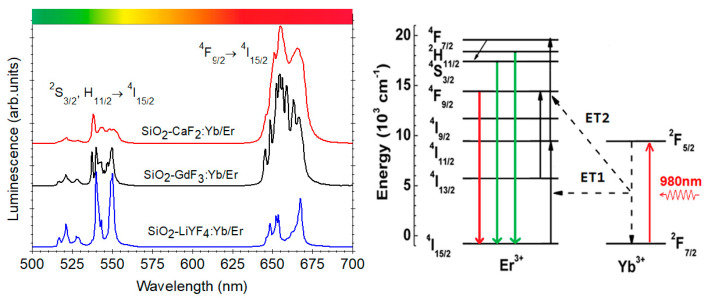
The green (^2^H_11/2_, ^4^S_3/2_ → ^4^I_15/2_) and red (^4^F_9/2_ → ^4^I_15/2_) Er^3+^ up-conversion luminescences excited at 980 nm in various Yb^3+^/Er^3+^-doped oxyfluoride glass ceramics [32] and the energy level schemes of Yb^3+^ and Er^3+^ with the main energy transfer processes.

A special case is represented by the up-conversion luminescence (UC) shown by the rare-earth ions (or ion pairs) that is an anti-Stokes luminescence process where synergistic effects of light excitation and mutual interactions between ions produce higher energy emission photons [31]. For rare-earth doped materials, the UC mechanism is based on the large absorption cross-section in the NIR region around 1000 nm of Yb^3+^ and a very efficient energy transfer (ET) to one of the Er^3+^/Ho^3+^ or Tm^3+^ ions. The absorption of infrared light photons by the Yb^3+^ ions (^2^*F*_7/2_ → ^2^*F*_5/2_ transition) is followed by a two-step energy transfer process to neighboring Er^3+^ ions with their characteristic *green* ((^2^*H*_11/2_, ^4^*S*_3/2_) → ^4^*I*_15/2_) and *red* (^4^*F*_9/2_ → ^4^*I*_15/2_) luminescent emissions (see Figure 6).

Nanocrystalline phase formation has a great effect on the optical response to ionizing radiation, and this can be characterized by using thermoluminescence measurement technique. Thermoluminescence (TL) represents the light emission by a solid sample during controlled heating after irradiation by ionizing radiation such as UV light, X-rays, gamma-rays, etc. [33] and it has proved to be a useful and sensitive tool for the study of radiation effects in various materials. According to the basic model, charge carriers (electrons and holes) produced by irradiation (stage a) are trapped in local energy levels *Tr*, (stage b), such as vacancies, interstitials, or impurities within the band gap; during the heating (stage c), they are thermally released and recombine at the recombination centers *R* giving rise to TL [33]; the thermal activation energy is *E*. In particular, TL has proved an effective tool for the study of rare-earth induced levels in material and their behavior as electron or hole trap [35,36,37].

**Figure 7:**
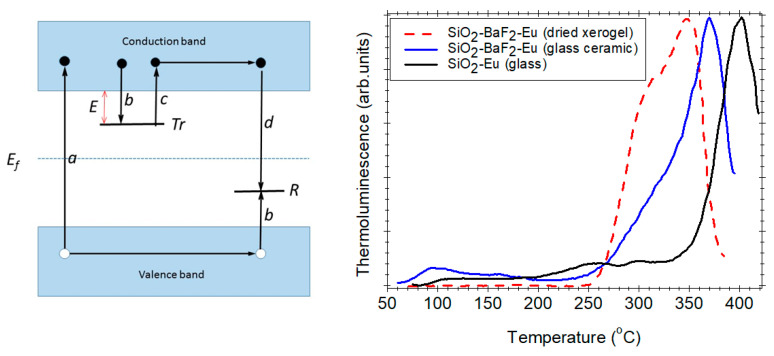
Thermoluminescence mechanism (**left**) (reproduced from reference [34]); and normalized glow curves recorded after X-ray irradiation at room temperature of Eu^3+^ doped SiO_2_–BaF_2_ dried xerogel and Eu^3+^ doped glass ceramic (prepared in ref. [8]) (**right**).

The TL properties of Eu^3+^-doped materials are due to the thermal release of the electrons from deep Eu^3+^-related traps followed by the recombination with the hole traps, resulting in the emission of light. TL properties were used to monitor the densification of Eu^3+^ doped SiO_2_–BaF_2_ dried xerogel prepared in ref. [8] to the glass ceramic (Figure 7-right). The investigations evidenced two glow peaks at 309 and 350 °C in the xerogel and a single dominant one at 370 °C in the glass ceramic, different from the glow peak observed in the Eu^3+^-silica glass at 400 °C [38]. This change was associated with structural changes during the ageing process of the gel and glass crystallization (Figure 7-right).

## 3. Oxyfluoride Glass Ceramic

In the following sections, we present the most relevant results about the optical properties (mainly photo- and up-conversion luminescence properties) for RE^3+^ doped oxy-fluoride glass ceramics, depending on the nature of the nanocrystalline phase.

### 3.1. SiO_2_–MeF_2_ Oxyfluoride Glass ceramic (with Me = Mg, Ca, Sr, Ba, Pb)

Alkali-earth fluorides are well-known optical materials with various applications for radiation detection (as scintillators and for thermoluminescence dosimetry) or as laser media due to excellent optical properties such as high transmittance from ultraviolet to mid infrared spectral range and easy incorporation of RE^3+^-ions.

#### 3.1.1. SiO_2_–CaF_2_ Oxyfluoride Glass Ceramic

The first report on 1 mol % ErF_3_-doped SiO_2_–CaF_2_ glass ceramic was by Zhou et al. [39]—the precipitation of CaF_2_ crystals of about 20 nm size, homogeneously distributed in the amorphous SiO_2_ matrix, is accompanied by the Er^3+^ related *red* (^4^*F*_9/2_ → ^4^*I*_15/2_) up-conversion luminescence. Further and deeper investigations on the UC luminescence properties of the Yb/Er doped SiO_2_–CaF_2_ glass ceramic and the mechanism involved were conducted by Georgescu et al. (2013) [40]. The *green* ((^2^*H*_11/2_, ^4^*S*_3/2_) → ^4^*I*_15/2_) and *red* (^4^*F*_9/2_ → ^4^*I*_15/2_) up conversion luminescences (Figure 6) are accompanied by weaker blue-UV luminescences at 380 nm (^4^*G*_11/2_ → ^4^*I*_15/2_) and 405 nm (^2^*H*_9/2_ → ^4^*I*_15/2_) assigned to the Er^3+^ ions deexcitation from higher energy levels. The analysis of the upconversion emissions indicated a two-photon processes for the red–green luminescence while the blue-UV luminescences is ascribed to three-photon processes. Their internal quantum efficiencies—0.88% (blue), 0.44% (green), and 10.6% (red)—were estimated from the fluorescence lifetimes of the blue, green, and red luminescences.

**Figure 8:**
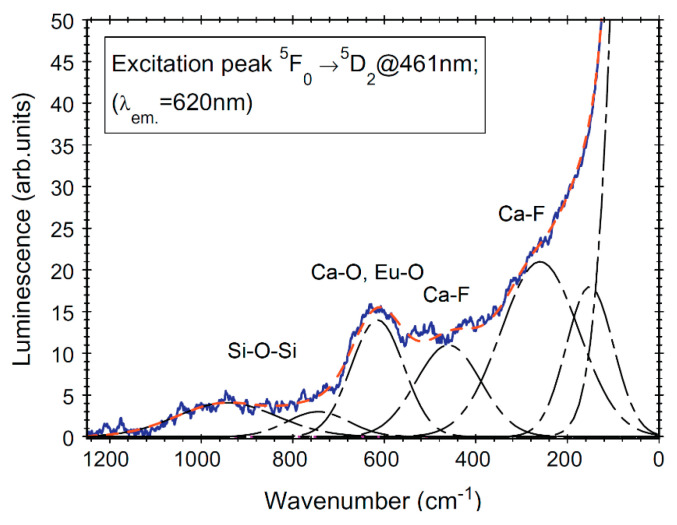
The PSB spectrum associated to the ^5^F_0_ → ^5^D_2_ transition of Eu^3+^-ion (at 461 nm), recorded in glass–ceramic (solid curve) and the fit with Gaussian-type curves (dashed curves); the abscissa is taken as the energy shift from the pure electronic transition (PET) peak ^5^F_0_ → ^5^D_2_ at 461 nm. (Modified from reference [15].)

The Eu^3+^ ions environment in the silica glass and SiO_2_–CaF_2_ glass ceramic was investigated by using phonon side bands (PSB) measurements [15,17] (Figure 8). The local structures around Eu^3+^ ions give rise to local vibration modes that can be observed as vibronic lines associated to excitation peaks, i.e., PSB. The PSB peaks were assigned to the vibrations of the SiO_4_ tetrahedra units of the glass network (above ≅500 cm^−1^) and Ca–F bonds vibrations in the precipitated CaF_2_ nanocrystalline phase (below ≅500 cm^−1^). The presence of Si–O, Eu–O, and Eu–F bonds is consistent with the Eu^3+^ ions partition in both silica glass matrix and CaF_2_ nanocrystaline phase, i.e., in the non-centrosymmetric sites of the CaF_2_ nanocrystals structure [15].

New evidence on the Eu^3+^ ions partition was provided by the thermoluminescence measurements. Thermoluminescence investigations of SiO_2_–CaF_2_ glass ceramic revealed a glow peak at 370 °C, assigned to the recombination of the electrons released from the Eu^3+^-electron traps in the CaF_2_ nanocrystals, which is shifted from the peak observed in the Eu^3+^-doped silica glass at 400 °C [38]; the broadening of the glow peaks is consistent with multiple Eu^3+^-ion sites.

#### 3.1.2. SiO_2_–SrF_2_ Oxyfluoride Glass Ceramic

**Figure 9:**
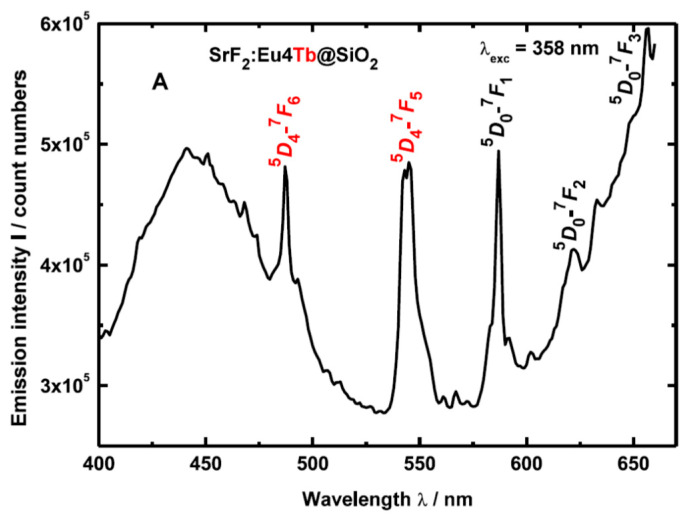
Photoluminescence spectra recorded on SrF_2_:Eu/Tb@SiO_2_ glass ceramics after under 368 nm excitation wavelength; blue broad emission of SiO_2_ is accompanied by the green light peaks of Tb^3+^ and red light peaks of Eu^3+^ (reproduced from reference [41]).

Luminescence properties of Eu^3+^/Tb^3+^ co-doped SiO_2_–SrF_2_ glass ceramics have been studied and showed cross-relaxation in materials with Tb^3+^ dopant as well as energy transfer from Tb^3+^ to Eu^3+^. White luminescence can be achieved by combining blue emission of SiO_2_, green light emitted by Tb^3+^ and red one by Eu^3+^, resulting in a white phosphor-like behavior [41] (Figure 9).

Up-conversion luminescence spectra of Er^3+^-doped SiO_2_–SrF_2_ oxyfluoride glass ceramic showed resolved Stark components of ^4^I_13/2_ band in the glass ceramics compared to the xerogel, assigned to the change of the environment around Er^3+^ from the glass to nanocrystals [42]. Moreover, the introduction of Al^3+^ to the SiO_2_ network caused an improvement of the optical properties: better transparency in the UV region due to lower pore content and intense visible UC luminescence as compared to the one with only SiO_2_ [42].

#### 3.1.3. SiO_2_–BaF_2_ Oxyfluoride Glass Ceramic

Transparent glass ceramics containing Er^3+^-doped BaF_2_ nano-crystals doped were prepared by sol–gel route and upconversion luminescence was assigned to the Er^3+^-ions incorporated within the BaF_2_ nanocrystals of about 2–15 nm size [43]. Further investigations of RE^3+^-doped SiO_2_–BaF_2_ glass ceramic (RE = Ho, Dy, Eu, Sm) showed that in the glass ceramic material, a large fraction of RE^3+^ optically active ions is partitioned into BaF_2_ nanocrystals of about 10 nm size [8,19]. The Eu^3+^-luminescence signal enhancement is accompanied by an increase of luminescence lifetime, from 0.27 ms in dried xerogel to 4.7 ms in the glass ceramic [8].

**Figure 10:**
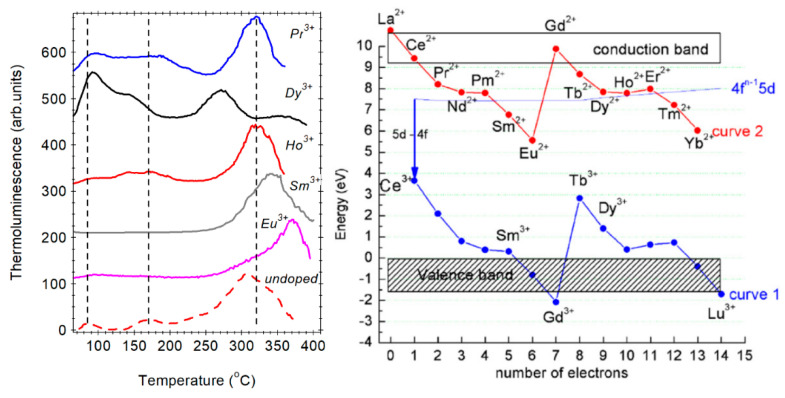
TL curves recorded on undoped (dotted line) and RE^3+^-doped glass ceramics (solid lines) after X-ray irradiation at room temperature (reproduced from reference [44]); the energy levels scheme of lanthanides in YPO_4_ (reproduced from reference [34]).

Thermoluminescence measurements recorded after X-ray irradiation of RE^3+^-doped SiO_2_–BaF_2_ glass ceramic indicated that new deep trap levels are introduced by the RE^3+^-doping: ≅140 °C (for Ho^3+^, Dy^3+^), 340 °C (for Sm^3+^), and 370 °C (for Eu^3+^); in undoped glass ceramic, the TL peak was observed at 383 °C. The glow peaks were assigned to the recombination of RE^3+^-related electron traps located mainly inside the BaF_2_ nanocrystals (Figure 10). Within the series, the trivalent lanthanide ions act as increasingly deeper electron trapping centers [34,35,36,37,38] and this can be observed as glow peaks shift in the temperature scale [36]. The glow peaks energy within the energy levels model proposed for the crystals might be influenced by the nanosize-related effects on the band gap energy.

#### 3.1.4. SiO_2_–PbF_2_ Oxyfluoride Glass Ceramic

The first study on Er^3+^-doped SiO_2_–PbF_2_ oxyfluoride glass ceramic made by Luo et al. [44] assigned the crystallization of β-PbF_2_ phase to a diffusion-controlled process of three-dimensional growth with decreasing nucleation. It was assumed that Er^3+^-ions segregated at the surface of the crystallites and hindered the growth of β-PbF_2_, thus postponing the crystallization. Later on, the studies were extended to crystallization behavior, structural investigation, and optical properties of other rare earth doped SiO_2_–PbF_2_ oxyfluoride glass ceramics.

Glass ceramic containing Eu^3+^/Tb^3+^ singly-doped β-PbF_2_ nanocrystals containing nanocrystals of around 10–15 nm size embedded in silica amorphous hosts were synthesized and their optical properties studied [12,45,46]. Luminescence decay kinetics showed non-exponential decays with shorter and longer luminescence lifetimes: (Eu^3+^: τ_1_(^5^D_0_) = 0.90 ms, τ_2_(^5^D_0_) = 5.15 ms; Tb^3+^: τ_1_(^5^D_4_) = 0.48 ms, τ_2_(^5^D_4_) = 4.01 ms) corresponding to two different surroundings around Eu^3+^ and Tb^3+^ dopants, silica glassy hosts, and β-PbF_2_ nanocrystals. The analysis of the luminescence intensity ratios as well as double-exponential character of luminescence decay curves clearly indicated the incorporation of RE^3+^ dopant ions into formed low phonon β-PbF_2_ nanocrystalline phase.

**Figure 11:**
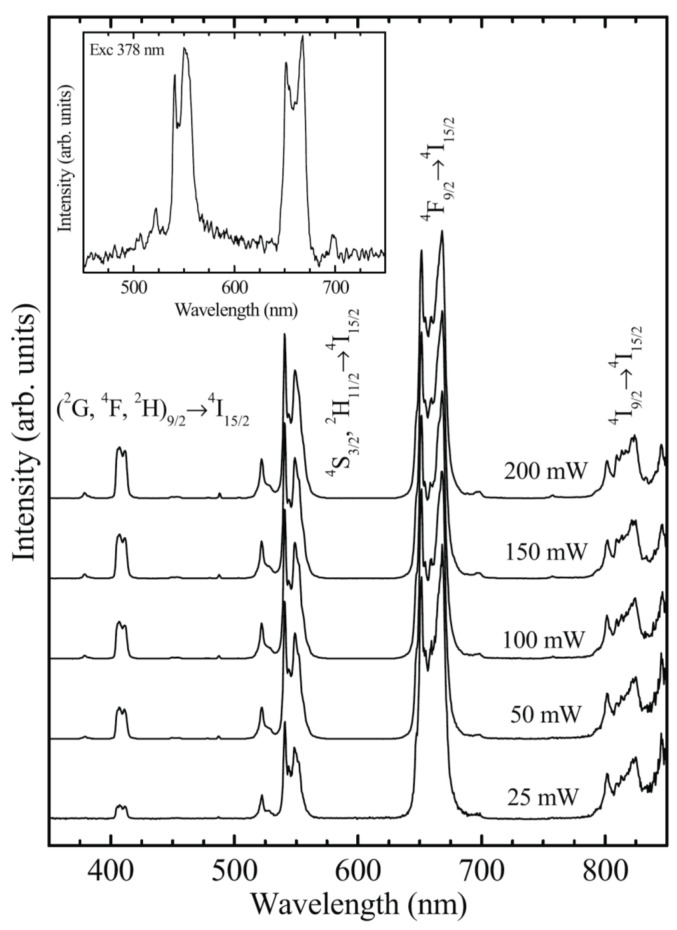
Up-conversion emission spectra of Er^3+^-doped SiO_2_–PbF_2_ glass ceramic under 980 nm IR light pumping at different powers; the inset shows emission spectrum excited at 378 nm (reproduced from reference [47]).

The up-conversions properties of transparent 0.3Yb^3+^/0.1Er^3+^ (mol%) co-doped 90SiO_2_–10PbF_2_ oxyfluoride glass–ceramics were extensively studied by J. del-Castillo et al. [47,48]. X-ray diffraction and electron microscopy analysis showed the precipitation of cubic β-PbF_2_ nanocrystals varying from 5 to 25 nm depending on heat treatment conditions at low temperatures, i.e., 300–400 °C. The up-conversion luminescence spectra showed *green* ((^2^*H*_11/2_, ^4^*S*_3/2_) → ^4^*I*_15/2_) and *red* (^4^*F*_9/2_ → ^4^*I*_15/2_) luminescence at 520–540 and 660 nm, respectively, accompanied by weaker blue luminescence at 410 nm (^2^*H*_9/2_ → ^4^*I*_15/2_) all assigned to the Er^3+^ transitions; the Stark splitting is consistent with the ions incorporation within the nanocrystals that assure an efficient energy transfer between ions (Figure 11).

The analysis of the dynamics of the up-conversion emissions indicated that distinct energy transfer excitation pathways are responsible for populating the luminescent levels: two and three photon processes for red-visible emission at 520–540 nm and 660 nm, and blue emission at 410 nm, respectively. Moreover, white up-conversion luminescence was obtained by combining the blue and red upconversion emissions band of Tm^3+^ with the upconversion green emission bands of Er^3+^ or Ho^3+^ and by using different pump powers and ratios between co-dopants [48].

### 3.2. SiO_2_–MeF_3_ Oxyfluoride Glass Ceramic (with Me = La, Y, Gd, Ce)

Rare-earth doped trifluorides and the corresponding oxyfluoride glass ceramics are attractive optical materials because of their wide band-gap, high solubility of rare-earth ions without additional charge compensation, and possible energy transfer (ET) processes to other co-dopant ions (for Gd^3+^ and Ce^3+^).

#### 3.2.1. SiO_2_–LaF_3_ Oxyfluoride Glass Ceramic

**Figure 12:**
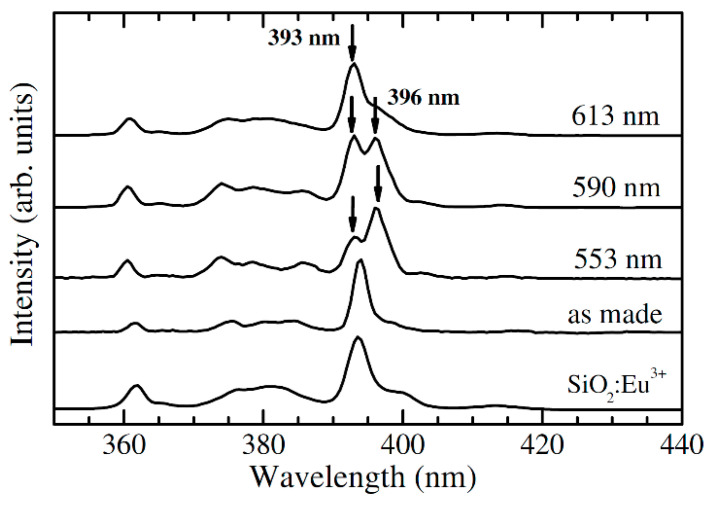
Excitation spectra of Eu^3+^-doped LaF_3_–SiO_2_ glass ceramics recorded at indicated wavelengths; the spectra of 89.9SiO_2_–10LaF_3_–0.1EuF_3_ glassy sample and SiO_2_:Eu^3+^ sol–gel glass detected at 590 nm are also included (reproduced from reference [49]).

The first studies on the glass ceramization investigations in the SiO_2_–LaF_3_ system revealed the importance of controlling the synthesis and heat treatment parameters [5,6,7]. Recent investigations on the crystallization process of SiO_2_–LaF_3_ glass–ceramics indicated a chemical reaction, followed by the fast precipitation of LaF_3_ crystals and not diffusion-controlled nucleation and growing process, as in the melt-quenched glasses [10]. Structural and optical investigations of nano-structured Eu^3+^-doped SiO_2_–LaF_3_ transparent glass–ceramics calcinated at 800 °C [49] were used to investigate the Eu^3+^ ions partition in both glassy and crystalline phases. Site selective luminescence spectroscopy revealed the 393 nm excitation peak assigned to the Eu^3+^ ions in the silica glassy phase, whereas the 396 nm peak would correspond to Eu^3+^ ions partitioned into LaF_3_ nanocrystals (Figure 12). It is concluded that about half the Eu^3+^ ions are partitioned into fluoride nanocrystals, while the rest remains in the glassy phase.

The liquid sol transformation to xerogel and then Eu-doped SiO_2_–LaF_3_ glass ceramic was investigated by using photoluminescence spectroscopy [20,50,51]. The Eu^3+^ luminescence spectra and luminescence decay analysis showing a double-exponential character (with τ_1_(^5^D_0_) = 2.07 ms, τ_2_(^5^D_0_) = 8.07 ms and τ_1_(^5^D_0_) = 0.79 ms, τ_2_(^5^D_0_) = 9.76 ms for powders and glass ceramics, respectively) indicated the incorporation of optically active Eu^3+^ ions from amorphous silica framework into the low phonon energy LaF_3_ nanocrystalline phase. Moreover, the intensity ratio between the two visible emissions (^5^D_0_–^7^F_2_/^5^D_0_–^7^F_1_) was used to analyze the local symmetry around Eu^3+^ ions: the observation of the ratio value decrease is consistent with the ions’ incorporation within the LaF_3_ nanocrystals.

**Figure 13:**
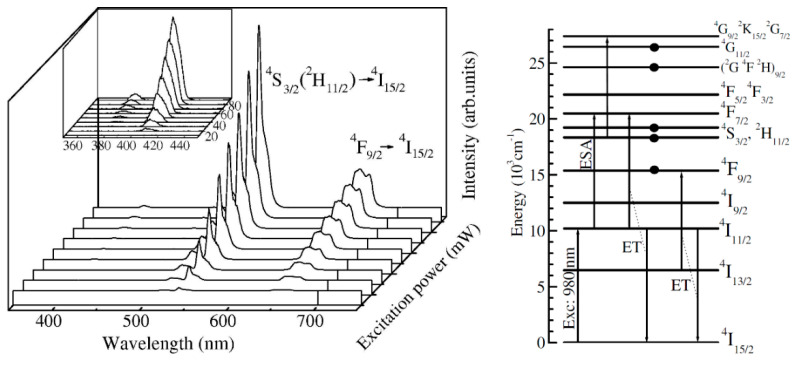
Up-conversion luminescence spectra of 95SiO_2_–5LaF_3_:0.1Er^3+^ glass ceramics recorded at room temperature under 980 nm IR light pumping with different pumping powers from 10 to 90mW (**left**) and the energy level schemes of Er^3+^ with the main energy transfer processes (**right**); reproduced from reference [52].

Up-conversion luminescence properties and the mechanism were investigated in 95SiO_2_–5LaF_3_:0.1Er^3+^ glass ceramic [52] (Figure 13) and Yb–Er or Yb–Ho, Yb–Tm doped SiO_2_–LaF_3_ glass ceramics [53,54,55,56]. Segregation of LaF_3_ nanocrystals in the matrix was confirmed by X-ray diffraction and upconversion emissions associated with the Er, Ho, and Tm ions in the nanocrystals were observed under 980 nm IR light excitation. Color tuneability and white light generation were observed in triple Yb^3+^/Ho^3+^/Tm^3+^ co-doped SiO_2_–LaF_3_ nano glass ceramics [57].

#### 3.2.2. SiO_2_–YF_3_ Oxyfluoride Glass Ceramic

Structural and morphological investigations of Eu^3+^-doped SiO_2_–YF_3_ sol–gel nano-glass ceramics have indicated the precipitation of YF_3_ nanocrystals with an average diameter of about 15 nm [57]. The reducing of the (^5^D_0_–^7^F_2_/^5^D_0_–^7^F_1_) intensity ratio value from 2.82 to 0.66 as well as bi-exponential character of decay curves (with shorter and longer lifetimes, 0.86 and 1.14 ms) was assigned to the incorporation of Eu^3+^ ions in both glassy and YF_3_ nanocrystalline phases, respectively.

An exhaustive analysis of the luminescence properties of (Eu^3+^, Sm^3+^) doped SiO_2_–YF_3_ structured glass–ceramic containing nanocrystals of around 11 nm was performed by A. Carlos Yanes et al. [23]. Selective excitation wavelengths and luminescence decay measurements allowed discerning between ions residing in precipitated YF_3_ nanocrystals and those remaining in a glassy environment; a large fraction of optically active ions is efficiently partitioned into nanocrystals. Moreover, for Yb^3+^–Tm^3+^ co-doped samples, bright and efficient up-conversion was observed, as well as very intense high-energy emissions in the UV range strongly dependent on the Yb^3+^ doping level opening the way to developing short-wavelength solid-state lasers for various photonic related applications [23]. The investigations of Tb^3+^/Eu^3+^ co-doped sol-gel glass ceramic materials containing MF_3_ (M = Y, La) nanocrystals showed Tb^3+^ → Eu^3+^ energy transfer process (ET) accompanied by multicolor emission due to the visible 4f^n^–4f^n^ transitions of Tb^3+^ and Eu^3+^ ions [58].

#### 3.2.3. SiO_2_–MeF_3_ Oxyfluoride Glass Ceramic (with Me = Gd, Ce)

Oxyfluoride glass ceramics containing GdF_3_ or CeF_3_ nanocrystals dispersed in the SiO_2_ matrix have been obtained and the optical properties of RE^3+^-ions studied. The energy levels overlap between the ^6^P_J_ states of Gd^3+^ and the UV-excited states of RE-ions promote an efficient energy transfer from Gd^3+^ to the RE^3+^ ions resulting in their characteristic photoluminescence. Crystalline phases analysis of RE^3+^-doped SiO_2_–GdF_3_ oxyfluoride glass ceramics has shown the precipitation of both hexagonal and orthorhombic GdF_3_ nanocrystals [59,60] or only orthorhombic one [61,62,63] in the silica matrix, depending on the synthesis path and the chemical composition of the system [50].

**Figure 14:**
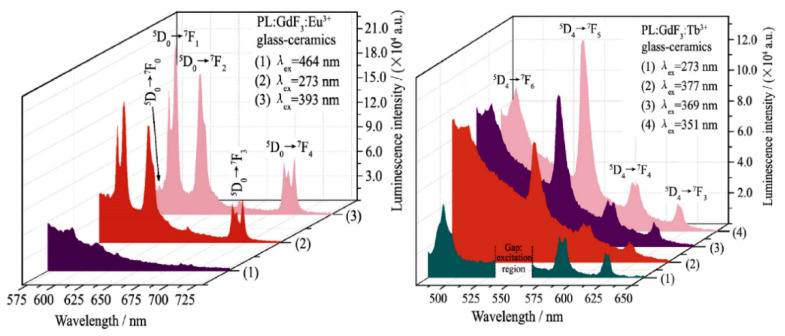
Photoluminescence spectra recorded in Eu^3+^ doped SiO_2_–GdF_3_ glass ceramic (**left**) and Tb^3+^ doped SiO_2_–GdF_3_ glass ceramic excited at Gd^3+^ or Eu^3+^/Tb^3+^ excitation wavelength peaks (reproduced from reference [63]).

The luminescence spectra and luminescence decay measurements recorded on Eu^3+^/Tb^3+^ doped SiO_2_–GdF_3_ oxyfluoride glass ceramics indicate the incorporation of the RE^3+^ ions within the nanocrystalline phase and silica glass matrix (Figure 14). The energy transfer process from Gd^3+^ to Eu^3+^ or Tb^3+^ ions was evidenced by the excitation spectra and luminescence spectra recorded under Gd^3+^ ions excitation wavelength at 273 nm [63].

**Figure 15:**
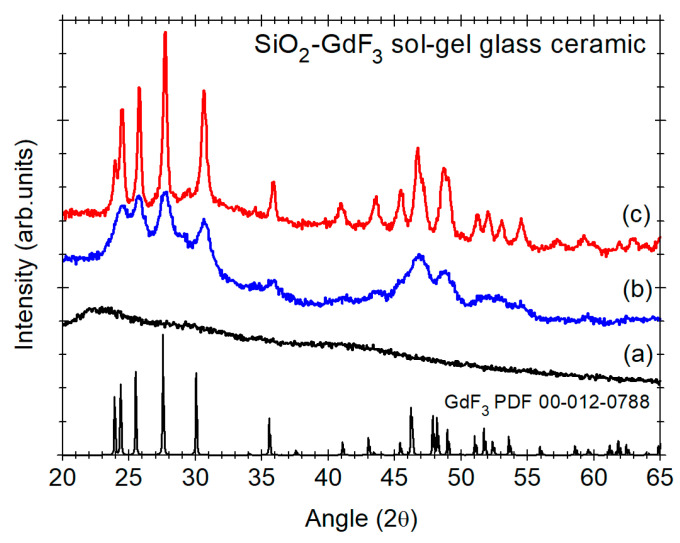
X-ray diffraction patterns of the Yb/Er co-doped SiO_2_–GdF_3_ xerogel (curve a) and glass ceramics undoped (curve b) and Li (1%) co-doped (c) [65]; the PDF file of orthorhombic GdF_3_ is shown for comparison.

Recently we used Fujuhara’s approach [5] for the synthesis of Eu-doped and Yb/Er co-doped SiO_2_–GdF_3_ and SiO_2_–LiGdF_4_ [64,65] glass ceramics and the key role played by the nature of the RE-dopant ions and Li co-dopants ions in the stabilization of orthorhombic GdF_3_ phase: the nanocrystals size increase from 9 nm to 25 nm is accompanied by strong lattice distortion evidenced by XRD peaks shift towards higher angles [65] (Figure 15). Under 980 nm IR light pumping, we observed up-conversion luminescence signal assigned to the Er^3+^ ions (Figure 6), with more than one order of magnitude higher in Li co-doped glass ceramic.

For RE^3+^-doped SiO_2_–CeF_3_ sol–gel glass–ceramics with RE = Eu, Sm, Yb/Er, structural and morphologic characterization has showed precipitation of the CeF_3_ nanocrystals [66]. The analysis of the intense red–orange emissions due to Eu^3+^ and Sm^3+^ transitions and the excitation spectra confirmed the partition of a large fraction of these ions into the precipitated CeF_3_ nanocrystals. A cross-relaxation process occurs between Eu^3+^ and Ce^3+^ ions and therefore the emissions from higher ^5^D_1_ and ^5^D_0_ energy levels are inhibited by the phonon-assisted energy transfer between the ions. In addition, in the Yb/Er co-doped samples, the presence of Ce^3+^ ions as a phonon-assisted cross-relaxation channel is responsible for a strong emission at 1.5 μm, which also leads to a drastic inhibition of the up-conversion emission.

## 4. Thernary and More Complex Oxyfluoride Glass Ceramic

The sol-gel route has been used to obtain other transparent glass ceramic compositions comprising thernary fluoride nanocrystalline Me1Me2F4 phases such as: SiO_2_–NaYF_4_ [67], SiO_2_–NaLaF_4_ [68], SiO_2_–NaGdF_4_ [69], SiO_2_–KLaF_4_ [70], SiO_2_–KYF_4_ [71], SiO_2_–LiYF_4_ [30,72,73] and their properties have been investigated. In these cases, the crystallization mechanism seems to be more complicated and the decomposition of metal trifluoroacetates is likely to be accompanied by some chemical reaction between metal and fluorine partners, followed by nanocrystalline phase precipitation within the glassy matrix. It was observed that the nature of the final precipitate crystalline phase is strongly dependent on the molar ratio between trivalent ion (Y, Gd, La) and alkali metals ions (Li, Na or K), and in general mixtures of fluorides were obtained (for LaF–NaLaF_4_ [68] and YF_3_–LiYF_4_ [30,72,73]) or different phase mixtures of the same compound (cubic and/or hexagonal NaGdF_4_ or KLaF_4_ nanocrystals [69,70]). Hence, an optimization of the initial composition and processing parameters (time and temperature) was required to obtain precipitation of the desired crystalline phase.

Optical properties of the Eu^3+^ ions were used to investigate local structure and xerogel to glass ceramic transformations in SiO_2_–NaYF_4_ [67] and SiO_2_–LiYF_4_ [30] glass ceramics. For Eu^3+^-doped SiO_2_–NaYF_4_ glass ceramics, cubic-NaYF_4_ nanocrystals of about 4–10 nm size precipitated during the thermal processing [67]. Site selective spectroscopy measurements allowed discerning ions in the amorphous silica glassy phase from those preferentially partitioned into a like-crystalline environment. Hence, a noticeable enhancement of the 613 nm luminescence from 3.1 to 5.7 ms by changing from amorphous surroundings (exciting at 464 nm) to those ions partitioned into nanocrystals (exciting at 392 nm) was observed. Moreover, the red to green emissions ratio (^5^D_0_–^7^F_2_/^5^D_0_–^7^F_1_) diminishes with the heat treatment indicating the partition of Eu^3+^ ions in the nanocrystals. In the SiO_2_–LiYF_4_ [72,73] glass ceramic, the precipitation of the LiYF_4_ nanocrystals was observed only for high Li excess (up to four molar Li/Y ratio). For a lower molar ratio, a glass–ceramic containing a mixture of YF_3_ and LiYF_4_ crystals or only YF_3_ phase (i.e., for stoichiometric ratio) was obtained [73]. The enhancement of Eu^3+^-lifetime values from 0.22 ms in the xerogel to 8.68 ms and the diminishing of the red to green emissions ratio were associated to the xerogel to glass ceramic transformation and Eu^3+^ ions partition in the nanocrystals [30]. Moreover, the group-theoretical analysis of the photoluminescence spectra has indicated that the Eu^3+^ ions incorporation occurs dominantly inside the LiYF_4_ nanocrystals with lower symmetry (C_2v_) sites than in polycrystalline pellet (D_2d_).

Transparent oxyfluoride glass–ceramics comprising Yb^3+^/Er^3+^ co-doped LiYF_4_ and NaYF_4_ nanocrystals showed green ((^2^*H*_11/2_, ^4^*S*_3/2_) → ^4^*I*_15/2_) and red (^4^*F*_9/2_ → ^4^*I*_15/2_) visible up-conversion luminescence due to the Er^3+^ ions incorporated in precipitated nanocrystals [73,74]. For Er/Yb co-doped SiO_2_–LiYF_4_ glass ceramic, the values of the quantum efficiencies for the red and green UC luminescences are much lower in the glass ceramic (*η* = 2% and *η* = 3.5%) compared to the pellet (*η* = 46% and *η* = 21%) [73], but similar to the Er/Yb co-doped glass ceramic containing CaF_2_ nanocrystals [40]. Moreover, the ratio between red and green up-conversion emission bands can be varied as a function of processing temperature and pump power resulting in color tunable up-conversion phosphors [74].

**Figure 16:**
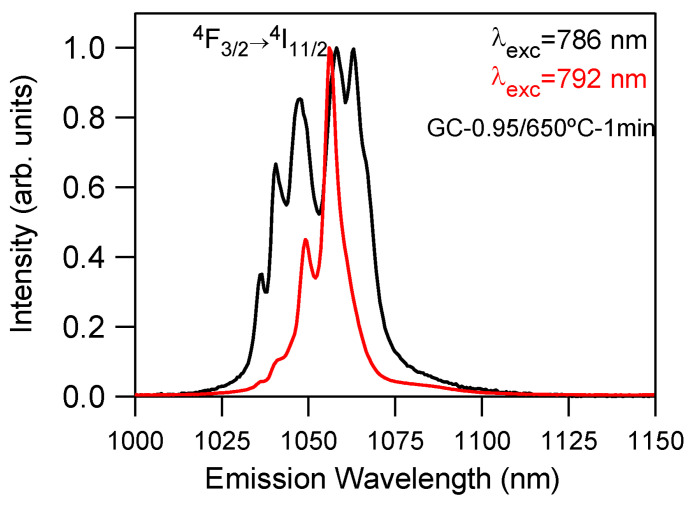
Normalized emission spectra of the ^4^F_3/2_ → ^4^I_11/2_ transition obtained under excitation at 786 (black) and 792 nm (red) of the GC-0.95 sample doped with 0.1 mol% of Nd^3+^ corresponding to Nd^3+^ ions in LaF_3_ and NaLaF_4_ crystalline phases, respectively [68].

The investigations of the Nd^3+^-doped SiO_2_–NaLaF_4_ glass ceramic showed both LaF_3_/NaLaF_4_ crystalline phases for different initial compositions and annealing temperatures; the crystallization of NaLaF_4_ was only promoted for Na deficiency in the precursors and higher temperatures calcination, above 650 °C [68]. The incorporation of Nd^3+^ ion into NaLaF_4_ and LaF_3_ nanocrystals was confirmed by site-selective emission and excitation spectra (Figure 16).

For the SiO_2_–NaGdF_4_ glass ceramic, the precipitation of cubic and/or hexagonal NaGdF_4_ nanocrystals with a size ranging between 4 and 24 nm was observed, depending on the Na:Gd ratio and processing conditions (temperature and time) [69]. The Na:Gd ratio was optimized to obtain the crystallization of β-NaGdF_4_ phase, more adequate for luminescent applications and for a molar ratio 0.95:1, the precipitation of β-NaGdF_4_ (JCPDS 027-0699) phase was observed after treatment at 550 °C. Luminescence results showed Eu^3+^ ions’ incorporation mainly in NaGdF_4_ NCs, and an efficient energy transfer Gd^3+^ to Eu^3+^ was observed. Electron microscopy investigations of SiO_2_–KLaF_4_ glass ceramics confirmed the coexistence of cubic (α-phase) and hexagonal (β-phase) KLaF_4_ phases, the last one being favored for high temperature calcination [70]. The spectral features of the Nd^3+^ ions dopants confirmed the incorporation of Nd^3+^ ions in both crystalline phases, with emission of Nd^3+^ predominantly in the β-KLaF_4_ hexagonal phase.

**Figure 17:**
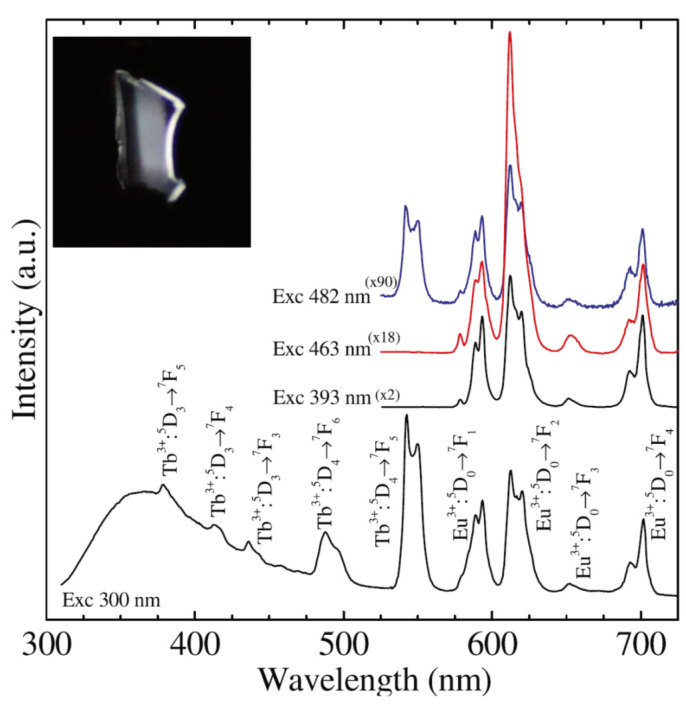
Emission spectra of 0.3Ce^3+^/0.3Tb^3+^/0.6Eu^3+^ (mol %) triply-doped SiO_2_–KYF_4_ glass ceramics under UV excitation of Ce^3+^ by comparison to the direct excitation of Eu^3+^ and Tb^3+^ ions, showing the Eu^3+^ and Tb^3+^ luminescence peaks (reproduced from reference [71]).

The UV light excitation of Ce^3+^/Tb^3+^/Eu^3+^ triply-doped SiO_2_–KYF_4_ glass ceramics is accompanied by an efficient ET processes between Ce^3+^ and Tb^3+^/Eu^3+^ ions, followed by their characteristic green and reddish-orange emission, respectively [71]. The emitted color can be tuned by varying the content of Eu^3+^ ions and/or the excitation wavelength, and white light generation was reached (Figure 17). In SiO_2_–KYF_4_ transparent glass–ceramics comprising Yb^3+^, Er^3+^, Tm^3+^ co-doped KYF_4_ nanocrystals, white light generation was obtained by simultaneous red, green, and blue additive up-conversion emissions of the RE^3+^-ions dopants [75].

More complex sol–gel glass ceramics, containing RE^3+^-doped BaYF_5_ and BaGdF_5_ nanocrystals embedded in silica matrix, have been produced by appropriate annealing of the corresponding precursor xerogel [76,77]. Optical measurements confirmed the distribution of a significant fraction of RE^3+^ ions into the precipitated BaYF_5_ fluoride nanocrystals environment [76]. For RE^3+^-doped (Eu^3+^, Sm^3+^, Dy^3+^ or Tb^3+^) glass ceramics containing BaGdF_5_ nanocrystals, intense visible emissions through efficient energy transfer from Gd^3+^ to RE^3+^ ions were observed, which leads to consider these materials as potential emitting phosphors for color converted UV LED [77]. Sol-gel glass ceramics comprising Tm^3+^ co-doped SiO_2_–BaY_0.78−x_Gd_x_Yb_0.2_Tm_0.02_F_5_ (x = 0–0.78) nanocrystals showed UC luminescence in the NIR, visible, and UV range assigned to the Tm^3+^-dopant ions distributed into nanocrystalline environments [78]. The substitution of Y^3+^ by Gd^3+^ leads to a continuous decrease of the UV emission intensities due to an effective energy transfer between Tm^3+^ and Gd^3+^ ions.

As RE^3+^-doped Lu-based fluorides are investigated for their applications as scintillators due to high absorption cross-section for any kind of radiation, the sol-gel route has been used to prepare RE-doped SiO_2_–Sr_2_LuF_7_ glass ceramics and their structural and optical properties investigated [79]. The luminescent features of Eu^3+^ ions, used as structural probes, revealed the distribution of RE^3+^ ions in both glass matrix and fluoride nanocrystals. Under 980 nm laser excitation of Yb^3+^–Tm^3+^, Yb^3+^–Er^3+^ and Yb^3+^–Ho^3+^ co-doped glass ceramics, intense UV, vis and NIR up-conversion emissions were observed.

## 5. Oxychloride Glass Ceramic

Sol-gel glass ceramics containing Er^3+^-doped BaCl_2_ nanocrystals of about tens of nm size embedded in a silica matrix were prepared but by replacing TFA with trichloroacetic acid (CCl_3_COOH) [80]. The crystallization process relies on thermal decomposition of Ba-trichloroacetate at about 300 °C followed by subsequent growth into BaCl_2_-nanocrystals at 745 °C; Er^3+^-ions are incorporated during the nanocrystals’ growth. Under 810 nm laser light pumping, it shows green ((^2^*H*_11/2_, ^4^*S*_3/2_) → ^4^*I*_15/2_) and red (^4^*F*_9/2_ → ^4^*I*_15/2_) Er^3+^ up-conversion luminescences, much weaker (only about 10%) compared to NaYF_4_:Er^3+^ (18%). Glassy nanocomposites containing Eu^3+^-doped LaOCl nanocrystals of about tens of nm size embedded in a silica matrix were prepared with the sol–gel route using trichloroacetic acid (CCl_3_COOH) as a chlorination agent [81]. It was shown that the LaOCl nanocrystalline phase precipitation is the result of the lanthanum chloride hydrolytic and oxidative reactions. As the annealing temperature increases, nanocrystals grow up to tens of nm size and Eu^3+^-ions are gradually incorporated inside the LaOCl nanocrystals, with a (*C*_4v_) local coordination symmetry.

## 6. Perspectives and Applications

In summary, the synthesis and investigations of sol-gel derived oxyfluoride glass ceramics are based and limited to the synthesis initially developed by Fujihara et al. [5,6,7], using TMOS and/or TEOS as the SiO_2_ precursor and TFA as the fluorine source and active fluoride crystalline phase fraction of about 5%, much lower than in melt-quenched glass ceramics. Most of the research efforts were dedicated to oxyfluoride glass ceramics, being focused primarily on optical (luminescence) properties of the RE-doped nanocrystals. Therefore, future research efforts are expected to be dedicated to the discovery of new glass ceramic materials with multifunctional properties (optical, electric, and magnetic) for new and improved applications.

Transparent glass ceramic waveguides offer specific characteristics of capital importance in photonics and the sol-gel approach has proved to be a very convenient and flexible way to deposit glass ceramic thin films on a variety of substrates for different applications, such as planar waveguides or integrated optics. New glass ceramic thin films comprising RE-doped oxide semiconducting nanocrystals such as SnO_2_, ZrO_2_, CeO_2_, HfO_2_ have been already obtained and optical properties studied [82,83,84,85,86]. The investigations of both optical and electric properties would be interesting for both basic and applied research.

New developments of the glass ceramic films can include patterned glass ceramic thin films that can be produced by using laser-induced crystallization [87] allowing the development of active integrated optical circuits. Moreover, a higher crystallized fraction of the nominal active fluoride crystalline phase is expected to improve the optical properties. Recent studies have shown that crack-free SiO_2_–LaF_3_ glass ceramic films with a crystalline LaF_3_ crystalline fraction of 18 wt% can be obtained [18].

The investigations of glass ceramics with Gd^3+^-based fluorides nanocrystals embedded in the glass matrix were limited to the analysis of luminescence properties of the co-dopant RE-ions [61,62,63]. The magnetic properties related to Gd^3+^ ions and the influence of the magnetic field on optical properties, in particular on the energy transfer processes, have been overlooked/missed. Such novel, multifunctional magneto-optical materials allow the intertwining between the magnetism and photonics and might offer new opportunities for magneto-optical devices.

Sol–gel synthesis has been used to obtain not only thin films but also monolithic silica and glass ceramics [88,89] opening the opportunity to produce monolithic scintillating glass ceramics based on BaF_2_, CaF_2_, CeF_3_ and BaCl_2_. Proper co-doping with broad blue absorbing sensitizer ions (Bi^3+^, Sn^3+^, etc.) capable to transfer their excitation energy to neighboring activator ions might develop potential spectral down-conversion applications.

The optical properties investigations of glass ceramics were focused only on trivalent ions related ones and divalent Eu^2+^ and Sm^2+^ ions were omitted. Previous investigations [90,91] have shown the incorporation of the reduced Eu^2+^ and Sm^2+^ ions in sol-gel glasses (not ceramic ones) under moderate conditions of temperature and atmosphere in two steps, glass-formation, and their reduction to the bivalent state by calcination in reducing atmosphere. Eu^2+^ and Sm^2+^ ions have attracted significant attention because they have a great potential for various photonics-related applications or radiation detection. The Sm^2+^ ion shows persistent spectral hole burning (PSHB) and potential application for high density optical memories. On the other hand, Eu^2+^ doping is crucial for scintillators detectors or X-ray storage phosphor for digital imaging applications. However, the incorporation of such bivalent ions into the precipitated nanocrystals from the glass ceramics still remain an open problem.

A new approach for sol-gel glass ceramics was recently proposed by Cruz M.E. et al. (2020) [92], where dispersed nanocrystals were incorporated in the silica glass matrix. This approach allows the incorporation in the silica glass matrix of a much broader range of nanocrystalline phosphors such as Eu^2+^-doped persistent phosphors [93] or Sm^3+^-doped BaFCl for multilevel optical data storage applications [94].

As the optical properties are strongly influenced by the hydroxyl ions (present even after high temperatures calcination), new non-aqueous sol-gel approaches are highly desirable [95,96]. A first step was made by using hydrofluoric acid catalyzed sol–gel process [95] and the non-aqueous fluorolytic sol–gel synthesis of metal nano-fluorides [96].

## 7. Conclusions

In conclusion, sol-gel derived glass ceramic materials based on stabilized rare-earth doped nanoparticles embedded in a glass matrix were demonstrated as novel, attractive materials for photonics applications. However, the advantages of sol-gel chemistry and thin films’ deposition ability have not been fully exploited for advances in both basic and applied research. Therefore, the development of new glass ceramic materials with multifunctional properties for new and improved applications is highly desirable. 

## Abbreviations

DSC	differential scanning calorimetry
ET	energy transfer
FTIR	Fourier transform infrared spectroscopy
IR	Infrared
NIR	near infrared
PL	photoluminescence
PSB	phonon side bands
PSHB	persistent spectral hole burning
RE	rare earth
TEOS	Tetraethyl orthosilicate, formally named tetraethoxysilane
TFA	trifluoroacetic acid
TG	thermogravimetry
TEM	Transmission electronmicroscopy
TL	Thermoluminescence
TMOS	Tetramethyl orthosilicate
UC	up-conversion
UV	ultraviolet
XRD	X-ray diffraction
